# Forced sterilization of women as discrimination

**DOI:** 10.1186/s40985-017-0060-9

**Published:** 2017-07-14

**Authors:** Priti Patel

**Affiliations:** 38 Beaumont Drive, Rolleston, 7614 New Zealand

**Keywords:** Sexual and reproductive rights, Sterilization, Women’s rights, Discrimination, Human rights, Patient care, Human rights in patient care

## Abstract

There has been a long history of subjecting marginalized women to forced and coerced sterilization. In recent years, the practice has been documented in countries in North and South America, Europe, Asia, and Africa. It has targeted women who are ethnic and racial minorities, women with disabilities, women living with HIV, and poor women.

A handful of courts have issued decisions on the recent forced sterilization of marginalized women finding that such actions violate the women’s rights. However, they have all failed to address the women’s claims of discrimination. The failure to acknowledge that forced sterilization is at its core a violation of the prohibition of discrimination undermines efforts to eradicate the practice. It further fails to recognize that coerced and forced sterilization fundamentally seeks to deny women deemed as “unworthy” the ability to procreate.

Four key principles outlined in the human rights in patient care framework highlight the importance of a finding that the prohibition of discrimination was violated in cases of forced sterilization: the need to highlight the vulnerability of marginalized populations to discrimination in health care settings; the importance of the rights of medical providers; the role of the state in addressing systemic human rights violations in health care settings; and the application of human rights to patient care.

Based on these principles, it is clear that finding a violation of the prohibition of discrimination in forced sterilization cases is critical in addressing the systemic nature of the practice, acknowledging the marginalization of specific groups and effectively ending forced sterilization through addressing the underlying purpose of the practice. If litigators, non-governmental organizations and judicial officers are mindful of these principles when dealing with cases of forced sterilization, it is likely that they will be better able to eradicate forced sterilization.

## Background

There has been a long history of forced and coerced sterilization of women throughout the world. The practice targets marginalized populations, including people diagnosed with a mental illness or disabled persons, racial minorities, poor women, and people living with specific illnesses, such as epilepsy.^1^ Throughout the early 20th century, countries passed laws authorizing the coerced or forced sterilization of those they believed should not be permitted to procreate. In the USA, more than half of the 50 states passed laws permitting the sterilization of people diagnosed with a mental illness and disabled persons, criminals, persons with specific physical illnesses, such as epilepsy, Native Americans, and African-Americans [1]. From the 1930s through the 1980s, Japan, Canada, Sweden, Australia, Norway, Finland, Estonia, Slovakia, Switzerland, and Iceland all enacted laws providing for the coerced or forced sterilization of mentally disabled persons, racial minorities, alcoholics, and people with specific illnesses [2].

More recently, forced and coerced sterilization against marginalized women has been documented in countries in North and South America, Europe, Asia, and Africa, including Chile, Czech Republic, Dominican Republic, Hungary, India, Kenya, Mexico, Namibia, Slovakia, South Africa, Swaziland, USA, Uzbekistan, and Venezuela [3].

The recent cases of forced and coerced sterilization target women living with HIV, women who are ethnic and racial minorities, women with disabilities, and poor women, among others [3]. The force or coercion has primarily taken the following forms [3–5]:

- *The women’s consent was obtained under duress*. In such cases, women are asked to sign consent forms while in labour or on their way to the operating theatre or are told or given the impression that to obtain another medical procedure, such as an abortion or caesarian section, they had to consent to sterilization.
- The *consent was invalid* because women were asked to sign a consent form for sterilization without being provided with full and accurate information regarding the sterilization procedure.
- *The women’s consent was not obtained*. In such cases, women have never been asked if they want to be sterilized but are informed of their sterilization after having undergone a cesarean section. In some instances, women are unaware they have been sterilized until they try to access contraceptives and are then informed that they have been sterilized.

Coerced and forced sterilizations have often been justified by medical personnel as necessary for public health. For instance, in the early 20th century, medical personnel argued that forced and coerced sterilizations were needed to address hereditary and genetic defects. In the late 20th century, forced and coerced sterilizations were considered necessary to address overpopulation [6]. In the 1990s, forced and coerced sterilizations were carried out in Peru as part of a discriminatory public health program. More recently, forced and coerced sterilizations of Roma women has been justified by medical personnel as necessary for their own medical benefit.

Women who have been subjected to forced and coerced sterilization have approached courts in a number of countries, including Namibia, Kenya, Chile, and Slovakia, arguing that their coerced and forced sterilization violated a number of their guaranteed rights, including their right to family and freedom from discrimination and cruel, inhuman, and degrading treatment.^2^ In two jurisdictions—Namibia and the European Court of Human Rights^3^—courts found that the women’s rights had been violated. However, with respect to the specific claim that the coerced sterilization violated their right to be free from discrimination, both jurisdictions found either that there was no violation or that it was not necessary to separately examine the discrimination claim.

Using the human rights in patient care framework, this article argues that the failure of courts to acknowledge that the coerced or forced sterilization of marginalized women amounts to a violation of their right to be free from discrimination points to a misunderstanding of the nature of forced and coerced sterilization as targeting women specifically because they are from population groups deemed unworthy of procreation and will thus result in a failure to eradicate the practice.

This article will outline the relevant principles provided under the human rights in patient care framework and outline the international human rights implicated by coerced and forced sterilization. It will then briefly summarize the recent court decisions in Namibia and at the European Court of Human Rights on forced and coerced sterilization. The article will then draw on the relevant principles outlined in the human rights in patient care framework to discuss why finding a violation of discrimination is essential. Finally, the article will provide recommendations for courts, litigators, and non-governmental organizations when addressing discrimination claims in forced and coerced sterilization cases.

## Human rights in patient care framework

The human rights in patient care framework (HRPC) refers to the application of human rights principles to the context of patient care. It brings together the rights of both patients and providers and focuses on systemic issues and the role of the state [7]. HRPC is a useful framework for understanding the nature of forced and coerced sterilization and thus how best to address it to ensure that the practice ends and victims are properly provided with redress.

There are four particular concepts under HRPC which help in illuminating the best ways to end the practice of forced and coerced sterilization. First, HRPC highlights the particular vulnerability of marginalized population to discrimination in health care settings, and the framework “reveals issues of discrimination and social exclusion that often underlie abuse against patients” [7]. Forced and coerced sterilization primarily targets women who are perceived as inferior or unworthy of procreation. Forced and coerced sterilization of marginalized women is part of existing stigma and discrimination facing the marginalized population. For instance, in Eastern Europe, Roma women are subjected to severe stigma and discrimination not only in healthcare settings, but also in education and in housing, among others.

Second, HRPC acknowledges the importance of the rights of medical providers. Medical providers have rights to decent working conditions, freedom of association, and due process, among others. Within the context of forced and coerced sterilization, this means that individual medical providers should not be targeted by government when the cause of the forced and coerced sterilization is structural and not merely the act of one individual medical provider. Often countries may argue that cases of coerced and forced sterilization are due to medical negligence or malfeasance on the part of individual providers. However, when numerous cases of coerced and forced sterilizations are documented, the problem is unlikely to resolve itself without addressing the structural issues, such as policies around how informed consent is obtained and the reduction of stigma and discrimination against targeted populations.

Third, HRPC focuses on the role of the state in appropriate patient care and in addressing any violations of human rights in such settings. In the recent cases of forced and coerced sterilization of marginalized women, the state’s role in creating the conditions where such practices can and have occurred and in failing to take appropriate action when such cases have been reported are central to understanding and addressing forced and coerced sterilization.

Finally, the HRPC framework applies human rights guaranteed under international treaties to patient care. The content of those rights relevant to forced and coerced sterilization are discussed in more detail below in order to aid in the understanding of how coerced and forced sterilization can violate international human rights. The particular rights relevant to forced and coerced sterilization are the right to health; the right to information; the right to liberty and security of the person; the right to be free from torture and cruel, inhuman and degrading treatment; and the right to be free from discrimination and equality.

### The right to health

The right to health is guaranteed under the International Covenant on Economic, Social and Cultural Rights (ICESCR), Convention on the Rights of Persons with Disabilities (CRPD), and the Convention on the Rights of the Child [8–10]. Bodily autonomy is an integral part of the right to health. The Committee on Economic, Social and Cultural Rights (CESCR), tasked with determining the content and scope of the rights guaranteed under the ICESCR, has noted that the right to health includes the “right to control one’s health and body, including sexual and reproductive freedom, and the right to be free from interference, such as the right to be free from torture, non-consensual medical treatment and experimentation.” [11].

The Convention on the Elimination of All Forms of Discrimination Against Women (CEDAW) also guarantees women the right to adequate services for maternal health [12] and protects a woman’s right to reproductive choice under article 16. The Committee on the Elimination of Discrimination Against Women (CEDAW Committee), tasked with determining the content and scope of the rights guaranteed under CEDAW, has noted that the right to quality health care services includes an obligation on states to ensure that health services are accessible and acceptable [13]. Acceptable services are services which allow for reproductive choice and are delivered in a way that ensures that a woman gives her fully informed consent, respects her dignity, guarantees her confidentiality, and is sensitive to her needs and perspectives [13].

The CEDAW Committee has explicitly noted that countries should not permit forced or coerced sterilization [13]. The CEDAW Committee addressed the coerced sterilization of a Roma woman in *AS v Hungary* [14]. AS was rushed to the hospital while pregnant with heavy bleeding. At the hospital, the doctor found that AS would need a caesarian section to remove her baby as the baby was dead. She signed a consent form while on the operating table for her caesarian section and for sterilization. The consent for the sterilization was handwritten by the doctor. The CEDAW Committee found that the coerced sterilization violated AS’s right to health, among other rights. In particular, the CEDAW Committee found that AS had a right to “specific information on sterilization and alternative procedures for family planning in order to guard against such an intervention being carried out without her having made a fully informed choice.” [14] The CEDAW Committee pointed to the following facts in finding that AS did not receive all the appropriate information in a way in which she could understand and thus her informed consent was not obtained: AS was in a poor state of health when she arrived at the hospital; had to be prepared for surgery, sign consent documents, and underwent two medical procedures in 17 min; and did not understand the Latin term for sterilization which is what was used in the consent form; and the consent form was barely legible and handwritten [14].

### Right to information

The right to information, guaranteed under the International Covenant on Civil and Political Rights (ICCPR) and the CRPD, is closely linked with the exercise of other fundamental rights, including the right to health [9, 15]. In interpreting the right to health, the CESCR has stated that health facilities, goods, and services must be accessible and that this includes, among other things, the “right to seek, receive and impart information and ideas concerning health issues.” [11] The CESCR has also confirmed that countries have an obligation to ensure that health information provided by service providers is accurate. This includes requirements that information not be withheld or intentionally misrepresented, particularly to propagate the specific religious or cultural beliefs of individual health workers [11]. The CEDAW Committee affirmed the close link between the right to information and the right to health in *AS v Hungary* where it held that the failure to provide AS with necessary information for informed consent violated her rights [14].

### Right to liberty and security of person

The right to security of person, guaranteed under the ICCPR, includes the right to determine what happens to one’s body [15]. The United Nations Special Rapporteur on the right of everyone to the enjoyment of the highest attainable standard of physical and mental health (Special Rapporteur on the Right to Health) has expressed that “guaranteeing informed consent is a fundamental feature of respecting an individual’s autonomy, self-determination and human dignity in an appropriate continuum of voluntary health care services” [16].

### Right to be free from cruel, inhuman, and degrading treatment

The right to be free from cruel, inhuman, and degrading treatment is guaranteed under the ICCPR, CRPD, and the Convention against Torture and Other Cruel, Inhuman or Degrading Treatment or Punishment (CAT). Coerced and forced sterilization is a clear violation of this right. The Human Rights Committee, tasked with determining the content and scope of the rights guaranteed under the ICCPR, has noted that the purpose of the right is to protect both dignity and the physical and mental integrity of the individual from acts that cause not only physical but also mental suffering. It has further noted that the right protects individuals from cruel, inhuman, or degrading treatment in “medical institutions” [17].

The Committee Against Torture has recommended that countries take urgent measures to investigate promptly, impartially, thoroughly, and effectively all allegations of involuntary sterilization of women, prosecute and punish the perpetrators, and provide the victims with fair and adequate compensation [18]. The United Nations Special Rapporteur on torture and other cruel, inhuman, or degrading treatment or punishment has emphasized that forced sterilization of women may constitute torture or cruel or inhuman treatment [19].

### Right to non-discrimination and equality

The right to equality and to be free from discrimination is guaranteed in numerous international treaties. CEDAW prohibits discrimination against women in accessing healthcare services. CRPD prohibits discrimination on the basis of disability and, in particular, recognizes that women and girls with disability face multiple discrimination. The ICCPR and the ICESCR also prohibit discrimination on the basis of gender, sexual orientation, health status, and race, among others. The ICCPR also provides for the right to equality. Failure to provide health care services that only women need is a form of discrimination against women [13]. General Recommendation 19 of the CEDAW Committee states that “[d]iscrimination against women includes acts that inflict physical, mental or sexual harm or suffering, threats of such acts, coercion and other deprivations of liberty” [20].

Discrimination under international law is defined as

imply[ing] any distinction, exclusion, restriction or preference which is based on any ground such as race, colour, sex, language, religion, political or other opinion, national or social origin, property, birth or other status, and which has the purpose or effect of nullifying or impairing the recognition, enjoyment or exercise by all persons, on an equal footing, of all rights and freedoms [21].^4^

The prohibition applies to both direct and indirect discrimination. Direct discrimination is generally defined as “when an individual is treated less favourably than another person in a similar situation for a reason related to a prohibited ground” [22]. Indirect discrimination “refers to laws, policies or practices which appear neutral at face value, but have a disproportionate impact on the [] rights… as distinguished by prohibited grounds of discrimination.” [22] Finally, the ICCPR and ICESCR prohibit discrimination on the basis of race, sex, and health status, among others.

## Recent case law on sterilization

Very few judicial bodies have recently issued decisions on cases challenging the forced and coerced sterilization of marginalized women. Two of these bodies were the European Court of Human Rights (ECHR) and the Supreme Court of Namibia.^5^ The three cases decided by the ECHR were brought by five Roma women [23–25]. In the first of these cases, a Roma woman was sterilized when she delivered her second child via Caesarean section. During her pregnancy, she only met with a medical doctor once. While she was in labour, her medical notes indicated that she asked for sterilization. Her consent signature was shaky. She submitted that while she was in labour, she was told she would die if she had another child and thus felt she had to agree to sterilization. Similarly, in Namibia, three women living with HIV sued the government for their coerced sterilizations [26]. One of the women was 26 years old when she was sterilized. She had gone to the hospital in labour and was told she would need a caesarean section. While in labour, she was given consent forms to sign. She was unclear as to what she was signing. She only learned she had been sterilized when she sought contraception after her delivery.

In both jurisdictions, the courts found that the coerced and forced sterilization of marginalized women violated the law. In particular, the ECHR—in three separate cases—found that the forced and coerced sterilization of Roma women violated the right to private and family life and the right to be free from torture and inhuman or degrading treatment [23–25]. In Namibia, the Supreme Court found that the forced and coerced sterilization of HIV-positive women violated their rights [26, 27]. The Court did not specify the exact legal basis of its findings. They could include the common law right to personality, the constitutional rights to human dignity, the right to liberty, and the right to found a family. Often national courts, including Namibia’s courts, take into account international legal obligations and reasoning when determining the scope and nature of similar rights guaranteed at national level.

However, both jurisdictions rejected the women’s argument that forced and coerced sterilization violated their right to be free from discrimination on the basis of their gender and ethnicity or health status. The ECHR found that it was unnecessary to engage in a discrimination analysis, while the Supreme Court of Namibia found there was insufficient evidence to prove a claim of discrimination [23–27]. Neither jurisdiction engaged in a robust analysis of why it failed to find a violation of the prohibition of discrimination. Without further reasoning or clarification from the courts, it is difficult to understand the particular reasons for why the ECHR and the Supreme Court of Namibia were unwilling to find a violation of the prohibition of discrimination. For instance, though the Supreme Court of Namibia indicated there was insufficient evidence for a discrimination claim, it failed to provide any information or guidance on what type of evidence would be needed for such a claim. The ECHR did not even engage in an inquiry under the discrimination claim and failed to provide any further information for that except to note that since it had found a violation based on other rights, there was no need to assess whether the right to be free from discrimination had been violated. However, finding a violation of the prohibition of discrimination is critical to ending forced and coerced sterilizations.

## Why is finding a violation of the prohibition of discrimination important?

The HRPC framework highlights why a finding that the right to non-discrimination was violated is critical to understanding and addressing forced and coerced sterilization: it is important to address the broad systemic nature of the practice in many countries; it is important for acknowledging the marginalization of specific groups in society as the court decision can act as a response to discrimination in society; and it is important for addressing the purpose underlying the practice.

First, acknowledging or finding that the forced and coerced sterilization of the litigants was done because the women were of a specified group deserving of non-discrimination protection acknowledges the broad systemic nature of the practice rather than limiting it to an individual case. Judge Ljiljana Mijovic who dissented in *VC v Slovakia*, an ECHR case addressing the coerced sterilization of Roma women, highlighted why a finding that coerced sterilization violated the right to be free from discrimination was important to address the broad and systemic nature of the coerced sterilization [23]. Judge Mijovic stated:

Finding violations of [the right to be free from inhuman and degrading treatment and the right to respect for family and private life] alone in my opinion reduces this case to the individual level, whereas it is obvious that there was a general State policy of sterilisation of Roma women under the communist regime (governed by the 1972 Sterilisation Regulation), the effects of which continued to be felt up to the time of the facts giving rise to the present case. Additionally, and in order to illustrate that not many things had changed regarding State policy towards the Roma population, in its third report on Slovakia [the European Commission against Racism and Intolerance] stated that public opinion towards the Roma minority remained generally negative. Furthermore, [the European Commission against Racism and Intolerance] expressed particular concern about reports indicating that Roma women had been, on an ongoing basis, subjected to sterilisation in some hospitals without their full and informed consent. The fact that there are other cases of this kind pending before the Court reinforces my personal conviction that the sterilisations performed on Roma women were not of an accidental nature, but relics of a long-standing attitude towards the Roma minority in Slovakia. To my mind, the applicant was “marked out” and observed as a patient who had to be sterilised just because of her origin, since it was obvious that there were no medically relevant reasons for sterilising her [23].

Once a court finds that the sterilization is due to discriminatory practices, it can change the issue from one of a few bad incidents to one requiring structural reform. This can also assist in ensuring that the rights of medical personnel are also taken into account as in many of these cases, the forced or coerced sterilization is not an individualized decision made by one or two medical personnel but part of a broader systemic problem.

Second, a judicial finding that forced and coerced sterilization was in violation of the prohibition of discrimination is important for acknowledging and affirming the marginalization of specific groups in society. In *Namibia v LM and Others*, a case challenging the coerced sterilization of three HIV-positive women in Namibia, the Supreme Court of Namibia determined that there was no support for a finding that the coercive sterilization of the three women was due to discrimination based on their health status, but it noted that “the tenor of the [women’s] evidence strongly suggests that they believe that their HIV positive status was the primary reason for their sterilization” [27]. For the women who have been subjected to coerced and forced sterilization, an acknowledgement from a court that they were discriminated against due to their specific status affirms their experience and marginalization. It can also act as a response to the discrimination in society signaling to the general public that discrimination even if not overt on the basis of health status or ethnicity occurs and should be rejected.

Third, it is important for courts to find a violation of the right to be free from discrimination to address the underlying reasons for the practice in order to effectively address it. For example, in the case of Roma women challenging their coerced sterilizations, a finding on discrimination outlining the negative stereotypes facing Roma women in Europe has a direct bearing on how to end the practice. If the practice is primarily targeted at Roma women, then healthcare workers need particularized training directed at eradicating their misperceptions of Roma women, including the view that they are lazy, poor, and reliant on the state to take care of their many children [28]. These negative perceptions of Roma women directly contribute to the practice of coerced sterilization.

Further, the failure of courts to fully investigate the discriminatory nature of coerced and forced sterilization points to a misunderstanding of the fundamental nature of forced and coerced sterilization. Forced and coerced sterilization is inherently a discriminatory practice. The motivating reason for forced and coerced sterilizations is to deny specific populations the ability to procreate due to a perception that they are less than ideal members of society. Historically, the forced and coerced sterilization of specific groups, including mentally disabled women, poor women, and women of specific ethnic groups, stemmed from a belief that certain groups should not be permitted to procreate for the betterment of society. In *Buck v Bell*, Justice Oliver Wendall Holmes held that a law providing for the forced sterilization of individuals suffering from a mental disability or epilepsy did not violate the equal protection and due process clauses of the US Constitution [29]. In the decision, Judge Holmes stated, “It is better for all the world, if instead of waiting to execute degenerate offspring for crime, or to let them starve for their imbecility, society can prevent those who are manifestly unfit from continuing their kind.” [29].

Forced and coerced sterilization is seen as necessary for both the good of society and for the wellbeing of women from specific population groups. The failure of a court to acknowledge this means that the court fundamentally misunderstands the actual harm caused by coerced sterilization and who it targets.

## Recommendations

To adequately address claims of forced and coerced sterilization and in particular to make a claim that the right to be free from discrimination was violated, litigators and non-governmental organizations representing the interests of women who have been subjected to coerced and forced sterilization should do the following:

- Include a claim that the right to be free from discrimination was violated in their legal papers.
- Ensure the discrimination claim alleges discrimination on the basis of sex (if the facts support such a claim) and on any other basis such as health status or ethnic or racial affiliation.
- Provide the court with detailed legal arguments and factual evidence supporting the discrimination claim.
- Engage in documentation of other cases of forced and coerced sterilization.
- Use advocacy strategies, such as raising awareness among the general public and engaging the media on the harm of forced and coerced sterilization.
- Consider requesting the court to issue structural remedies to address the systemic issues of discrimination in healthcare.
- Ensure the remedies requested from the court reflect the desires of the affected women. For instance, it may be that the affected women prefer direct access to fertility services rather than mere monetary compensation.

Judicial officers when confronting cases of coerced or forced sterilization should do the following:

- Engage in a robust analysis of the discrimination claim.
- If the court determines there is insufficient evidence in the case at hand, then provide guidance on what type of additional evidence is needed.
- Consider structural remedies for addressing systemic discrimination in healthcare, including requiring the government to provide appropriate training of healthcare workers.
- Be open to considering granting alternative remedies to monetary compensation when appropriate and available.

In general, medical personnel, social workers, and community health workers should do the following:

- Ensure that healthcare workers are well trained on informed consent.
- Ensure healthcare workers are trained on how to work with marginalized populations to ensure they are not subjected to discriminatory treatment.
- Develop internal complaint processes such that any violations can be identified and appropriately addressed quickly and fairly.

## Conclusion

Recognizing that the forced and coerced sterilization of women is fundamentally a violation of the prohibition on discrimination is an essential step in ensuring the practice is ended. If litigators, non-governmental organizations, and judicial officers are mindful in the claims they bring and remedies they issue, it is likely that they will be better able to eradicate the practice.

## Abbreviations

CAT: Convention against Torture and Other Cruel, Inhuman or Degrading Treatment or Punishment
CEDAW: Convention on the Elimination of All Forms of Discrimination Against Women
CESCR: Committee on Economic, Social and Cultural Rights
CRPD: Convention on the Rights of Persons with Disabilities
ECHR: European Court of Human Rights
HRPC: Human rights in patient care
ICCPR: International Covenant on Civil and Political Rights
ICESCR: International Covenant on Economic, Social and Cultural Rights

## Footnotes

1 This paper uses the terms forced and coerced sterilization interchangeably to refer to sterilization without informed consent.

2 The case in Kenya is currently before the High Court awaiting a hearing date. The case in Chile is currently before the Inter-American Commission on Human Rights awaiting a decision. More information on the Kenya case can be found: www.kelinkenya.org. More information on the Chile case can be found: https://www.reproductiverights.org/lbs-fs-vs-chile.

3 The decisions of the European Court of Human Rights address forced and coerced sterilization in Slovakia.

4 CEDAW defines discrimination against women under article 1 as “any distinction, exclusion or restriction made on the basis of sex which has the effect or purpose of impairing or nullifying the recognition, enjoyment or exercise by women, irrespective of their marital status, on a basis of equality of men and women, of human rights and fundamental freedoms in the political, economic, social, cultural, civil or any other field” [12].

5 The CEDAW Committee’s decision in *AS v Hungary* is not included in this as it is a quasi-judicial body.

## References

[1] Peal TR. The continuing sterilization of undesirables in America. New Jersey: Rutgers Race and L. R; 2004;6:225.

[2] Alexander LL, Larosa JH, Bader H, Garfield S (2013). New dimensions in women’s health.

[3] Open Society Foundations. Against her will: forced and coerced sterilization of women worldwide. 2011. Available at: https://www.opensocietyfoundations.org/publications/against-her-will-forced-and-coerced-sterilization-women-worldwide. Accessed 30 Nov 2016.

[4] African Gender and Media Initiative (2012). Robbed of choice: forced and coerced sterilization experiences of women living with HIV in Kenya.

[5] International Community of Women Living with HIV/AIDS. The forced and coerced sterilization of HIV positive women in Namibia. March 2009. Available at: http://www.icw.org/files/The%20forced%20and%20coerced%20sterilization%20of%20HIV%20positive%20women%20in%20Namibia%2009.pdf. Accessed 30 Nov 2016.

[6] Stern AM (2005). Sterilized in the name of public health: race, immigration, and reproductive control in modern California. Am J Public Health.

[7] Cohen J, Ezer T (2013). Human rights in patient care: a theoretical and practical framework. Health Hum Rights.

[8] United Nations. 1976 International covenant on economic, social and cultural rights, art. 12. Available at: http://www.ohchr.org/EN/ProfessionalInterest/Pages/CESCR.aspx. Accessed 3 Jan 2017.

[9] United Nations. 2008 Convention on the rights of persons with disabilities. Available at http://www.ohchr.org/EN/HRBodies/CRPD/Pages/ConventionRightsPersonsWithDisabilities.aspx. Accessed 3 May 2017. 10.1515/9783110208856.203 18348362

[10] United Nations. 1990 Convention on the rights of the child, art. 24. Available at: http://www.ohchr.org/en/professionalinterest/pages/crc.aspx. Accessed 3 Jan 2017.

[11] Committee on Economic, Social and Cultural Rights (2000). General comment 14: the right to the highest attainable standard of health (Art. 12).

[12] United Nations. 1979 Convention on the elimination of all forms of discrimination against women. Available at: http://www.un.org/womenwatch/daw/cedaw/text/econvention.htm. Accessed 3 January 2017.

[13] Committee on the Elimination of Discrimination against Women (CEDAW). General recommendation no. 24: Article 12 of the convention (women and health). 1999. Available at: http://www.refworld.org/docid/453882a73.html. Accessed 30 Nov 2016.

[14] Communication No. 4/2004. CEDAW/C/36/D/4/2004. 2006. Available at: https://www.escr-net.org/sites/default/files/CEDAW_Committee_Decision_0.pdf. Accessed 30 Nov 2016 [hereinafter AS].

[15] United Nations. 1976 International Covenant on Civil and Political Rights (ICCPR). Available at: http://www.ohchr.org/en/professionalinterest/pages/ccpr.aspx. Accessed 3 Jan 2017.

[16] United Nations General Assembly Special Session. Report of the special rapporteur on the right to the enjoyment of the highest attainable standard of physical and mental health. 10 August 2009. para 18. Available at: http://www.refworld.org/pdfid/4aa762e30.pdf. Accessed 30 Nov 2016.

[17] Human Rights Committee. General comment no 20: Article 7 (prohibition of torture, or other cruel, inhuman or degrading treatment or punishment). 1992. Available at: http://www.refworld.org/docid/453883fb0.html. Accessed 30 Nov 2016.

[18] United Nations Committee Against Torture (CAT Committee). Concluding observations: Slovakia. 2009. para. 14. Available at: http://www.refworld.org/publisher,CAT,CONCOBSERVATIONS,SVK,4b66c9542,0.html. Accessed 30 Nov 2016.

[19] Human Rights Council. Report of the special rapporteur on torture and other cruel, inhuman or degrading treatment or punishment, Juan E. Méndez. 1 February 2013. Available at: http://www.refworld.org/docid/51136ae62.html. Accessed 30 Nov 2016.

[20] Committee on the Elimination of Discrimination against Women. General recommendation no. 19: violence against women. 1992. Available at: http://www.refworld.org/docid/52d920c54.html. Accessed 30 Nov 2016.

[21] Human Rights Committee. CCPR General comment no. 18: non-discrimination. 1989. Available at: http://www.refworld.org/docid/453883fa8.html. Accesssed 17 May 2017.

[22] Committee on Economic Social and Cultural Rights. General comment no. 20. Non-discrimination in economic, social and cultural rights. 2009. Available at: http://www.refworld.org/docid/4a60961f2.html. Accesssed 17 May 2017.

[23] VC v Slovakia. No. 18968/07. 2011.

[24] NB v Slovakia. No. 29518/10. 2012

[25] IG v Slovakia. No. 15966/04. 2013.

[26] LM and Others v Government of Namibia. Case Nos. 1603/2008; 3518/2008; 3007/2008. 30 July 2012.

[27] Government of Namibia v LM and Others. Case No. 49/2012. 3 Nov 2014 at para. 2.

[28] Center for Reproductive Rights. Body and soul: forced sterilization and other assaults on Roma reproductive freedom in Slovakia. 2003. Available at: https://www.reproductiverights.org/document/body-and-soul-forced-sterilization-and-other-assaults-on-roma-reproductive-freedom. Accessed 30 Nov 2016.

[29] Buck v Bell. 274 US 200. 1927.

